# PIAS3 promotes homology-directed repair and distal non-homologous end joining

**DOI:** 10.3892/ol.2013.1472

**Published:** 2013-07-17

**Authors:** SHICUI LIU, ZHONGYI FAN, ZHENGYING GENG, HAO ZHANG, QINONG YE, SHUNCHANG JIAO, XIAOJIE XU

**Affiliations:** 1 School of Medicine, Nankai University, Tianjin 300071, P.R. China; 2 Department of Oncology, Chinese PLA General Hospital, Beijing 100853, P.R. China; 3 Department of Medical Molecular Biology, Beijing Institute of Biotechnology, Beijing 100850, P.R. China

**Keywords:** PIAS, double-strand break repair, homologous recombination, non-homologous end joining

## Abstract

A DNA double-strand break (DSB) is the most severe form of DNA damage and is mainly repaired through homologous recombination (HR), which has a high fidelity, or non-homologous end joining (NHEJ), which is prone to errors. Defects in the DNA damage response lead to genomic instability and ultimately the predisposition of organs to cancer. Protein inhibitor of activated STAT-1 (PIAS1), which is a potential small ubiquitin-related modifier (SUMO) ligase, has been reported to be involved in DSB repair. The present study identified that another member of the PIAS family, PIAS3, is also an enhancer for HR- and NHEJ-mediated DSB repair. Furthermore, the overexpression of PIAS3 was demonstrated to increase the resistance of HeLa cells to ionizing radiation (IR), indicating a significant role for PIAS3 in the DNA damage response (DDR) pathway.

## Introduction

Cells are constantly exposed to varieties of genotoxic stress, including UV radiation, ionizing radiation (IR), chemical agents and reactive oxygen species, which induce potentially harmful DNA lesions (1). Human beings have evolved a highly efficient and complex system, the DNA damage response (DDR) pathway, to cope with damaged DNA (2). The DDR process includes cell cycle checkpoint activation to stop the cell cycle progression in order to allow time for DNA repair or apoptosis when the DNA damage is irreparable (3). Failure to properly sense and repair DNA may promote the accumulation of chromosomal rearrangements, which in turn fuels malignant transformation and finally leads to the occurrence of a tumor (4). Of the various forms of DNA damage, DNA double-strand breaks (DSBs) result in the most deleterious damage (5). One single DSB is sufficient to kill a mammalian cell.

In mammalian cells, DSBs are mainly repaired through non-homologous end joining (NHEJ), which is susceptible to errors, and homologous recombination (HR), which has a high fidelity (6). HR repair occurs in the S and G_2_ phases of the cell cycle due to its requirement of a homologous chain as a template to complete the repair process, whereas NHEJ repair joins the broken DNA together with no or simple processing of the ends of the DNA (7). Thus, HR-mediated and NHEJ-mediated DSB repair are essential for genome integrity.

The response to DSBs is initially detected by the Mre11-Rad50-Nbs1 (MRN) complex (8). In particular, cells activate the DDR protein kinases, ataxia telangiectasia mutated gene (ATM), ataxia telangiectasia and Rad3-related protein (ATR) and DNA-dependent protein kinase (DNA-PK; also known as PRKDC) (1). These then trigger histone H2AX phosphorylation and the accumulation of proteins, including MDC1, 53BP1, BRCA1, CtIP, RNF8 and RNF168/RIDDLIN, into ionizing radiation-induced foci (IRIF) that amplify DSB signaling and promote DSB repair (9). Following DSB formation, the attachment of a small ubiquitin-related modifier (SUMO) of the target proteins also accumulates at the DSB sites, which is a significant modification in the DDR pathway (10).

Protein inhibitors of activated STAT (PIAS) proteins are often identified to be associated with SUMO-modified substrates, further emphasizing their role as potential SUMO ligases (11). In this mode of function, the PIAS proteins are believed to act as adapter proteins that enhance the interactions between the SUMO conjugating enzyme, Ubc9, and the substrate proteins (12). In previous studies, PIAS1 has been established to recruit to damage sites and to promote DSB repair, indicating a significant role in the DDR pathway (13). However, the function of other PIAS members in DSB repair and which method of repair they are involved in remains largely unknown. The present study investigated whether another PIAS member, PIAS3 was involved in the components of the DDR and its actions at the DSB sites, in the processes of NHEJ or HR.

## Materials and methods

### Cell lines, plasmids and antibodies

The human 293T and HeLa cell lines were purchased from the American Type Culture Collection (ATCC; Rockville, MD, USA). The green fluorescent protein (GFP) reporter system for HR-mediated DSB repair direct repeat (DR)-GFP 293T cells, the GFP reporter system for NHEJ-mediated DSB repair EJ5-GFP 293T cells and the I-SceI expression construct were obtained from the City of Hope National Medical Center/Beckman Research Institute (Duarte, CA, USA). All the cell lines were cultured in Dulbecco’s modified Eagle’s medium (DMEM; Hyclone, Logan, UT, USA) with 10% fetal bovine serum (FBS; Hyclone) at 37°C in the presence of 5% CO_2_. The full-length coding sequences of PIAS1 and PIAS3 were amplified using PCR and cloned into a pXJ-40-myc vector. Hemagglutinin-tagged BRCA1 (HA-BRCA1) was constructed as previously described (14). The antibody for ATM, the myc-horseradish peroxidase (HRP), the HA-HRP and the HRP-conjugated secondary antibody were purchased from Sigma-Aldrich (St. Louis, MO, USA).

### HR- or NHEJ-mediated DSB repair GFP reporter systems

The HR-mediated DSB repair assay was performed as previously described (15). Briefly, DR-GFP 293T cells were delivered with ATM-RNAi (Lipofectamine; Invitrogen, Carlsbad, CA, USA) to the DR-GFP 293T cells using lipid-mediated transfection (RNAiMAX, Lipofectamine; Invitrogen) according to the specifications. Certain DR-GFP 293T cells were transfected with HA-BRCA1 or myc-PIAS1/3 using Lipofectamine 2000 (Invitrogen) according to the manufacturer’s instructions. At 24 h post-transfection, the cells were transfected with an I-SceI expression plasmid (pCBA Sce) using Jet Prime (Polyplus, Illkirch, France). Two days later, the GFP^+^ cells were assayed by FACScan (BD Biosciences, San Jose, CA, USA). The NHEJ-mediated DSB repair assays in the EJ5-GFP 293 cells were performed as previously described (15). Briefly, the EJ5-GFP 293 cells with the EJ5-GFP reporter stably integrated into their genome were transfected with HA-BRCA1 or myc-PIAS1/3. A second transfection was performed 24 h later with an empty vector or an I-SceI-expressing construct. Following the second transfection, the cells were harvested for 72 h and the fraction of the GFP^+^ cells was determined using flow cytometry (BD Biosciences).

### Immunoblotting

The total cell lysate was extracted with RIPA buffer and a protease inhibitor mixture (Roche, Basel, Switzerland). Precipitates or total cell lysates were resolved in 10% SDS-PAGE and transferred onto a nitrocellulose membrane. The blots on the nitrocellulose membrane were blocked using 5% skimmed milk in TBST (PBS with 0.05% Tween-20) and sequentially incubated with primary antibodies and HRP-conjugated secondary antibodies in 5% skimmed milk in TBST. The blots were washed with TBST following a 1-h incubation period. The immunoreactive bands were visualized using Peroxide Solution and Luminol/Enhancer Solution (Amersham Pharmacia, Amersham, UK).

### IR survival assay

The HeLa cells were transfected with myc-PIAS and empty vector and exposed to IR. The cells were left for 10–14 days at 37°C to allow colony formation. The colonies were stained with 0.5% crystal violet/20% ethanol and counted. The results were normalized to plating efficiencies.

## Results

### Establishment of the DR- and NHEJ-mediated DSB repair system

**Figure 1:**
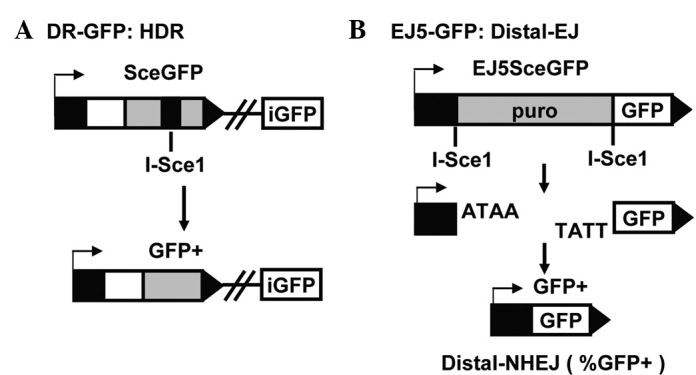
Establishment of the EJ5-GFP and DR-GFP systems. (A) DR-GFP is shown along with the HDR product that uses iGFP as the template for nascent DNA synthesis, which results in the restoration of a GFP expression cassette. (B) EJ5-GFP is shown along with products of EJ between the distal DSB ends (distal-EJ) that restores the GFP expression cassette. DR-GFP, direct repeatgreen fluoresecent protein; HDR, homology-directed repair; NHEJ, non-homologous end joining; DSB, double-strand break; iGFP, intense GFP.

**Figure 2:**
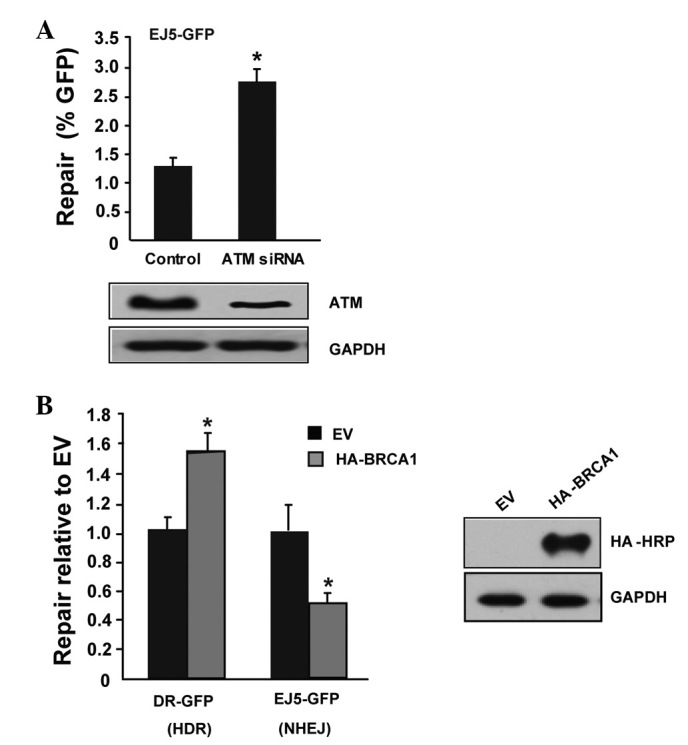
Testing the accuracy of EJ5-GFP and direct repeat (DR)-GFP systems using classical factors. (A) ATM specifically inhibits distal-NHEJ DSB repair. The GFP^+^ cell percentages were assayed using FACScan in the control and ATM-knockdown cells. ^*^P<0.01 vs. control cells. Western blot analysis of ATM expression in human 293T cells is shown in the lower panel. (B) Two individual cell lines were transfected with an expression vector for I-SceI, along with a complementation vector for BRCA1 or the empty expression vector (EV). Repair is measured as the percentage of GFP^+^ cells, which is normalized to the EV samples transfected in parallel. ^*^P<0.001 vs. EV. Representative western blots of BRCA1 expression in human 293T cells is shown in the right panel with BRCA1 carrying HA-HRP. ATM, ataxia telangiectasia mutated gene; NHEJ, non-homologous end joining; DSB, double-strand break; GFP, green fluorescent protein; HRP, horseradish peroxidase.

The HR-and NHEJ-mediated DSB repair systems were established to study the effect of PIAS3 in DSB repair. The reporter system that was stably integrated in the DR-GFP 293T cells was used to measure the HR-mediated DSB repair efficiency and the GFP-based chromosomal reporter EJ5-GFP in the 293T cells was used to measure the total NHEJ repair efficiency. DR-GFP was constructed using the homology-directed repair (HDR) product that uses intense GFP (iGFP) as the template for nascent DNA synthesis, which results in the restoration of a GFP expression cassette (Fig. 1A). EJ5-GFP contains a promoter that is separated from a GFP coding cassette by a puromycin gene flanked by two I-SceI sites in the same orientation. Once the puromycin gene is excised by the two I-SceI-induced DSBs, the promoter is joined to the rest of the expression cassette by NHEJ repair, leading to restoration of the GFP^+^ gene (Fig. 1B). Therefore, the number of GFP^+^ cells is a measure of the NHEJ-mediated DSB repair. To test the accuracy of the two systems, the present study utilized two factors with known functions that are involved in the DSB pathway to the systems, ATM and BRCA1. As expected, knockdown of ATM with specific ATM siRNA increased NHEJ-mediated DSB repair (Fig. 2A). Transfection of HA-BRCA1 increased the level of HDR and reduced the level of NHEJ-mediated DSB repair (Fig. 2B), which is consistent with a previous study (16). Taken together, the results of the tests of the two classical factors, ATM and BRCA1, indicated that the HR- and NHEJ-mediated DSB repair systems were established successfully.

### PIAS3 promotes HDR and distal-NHEJ

**Figure 3:**
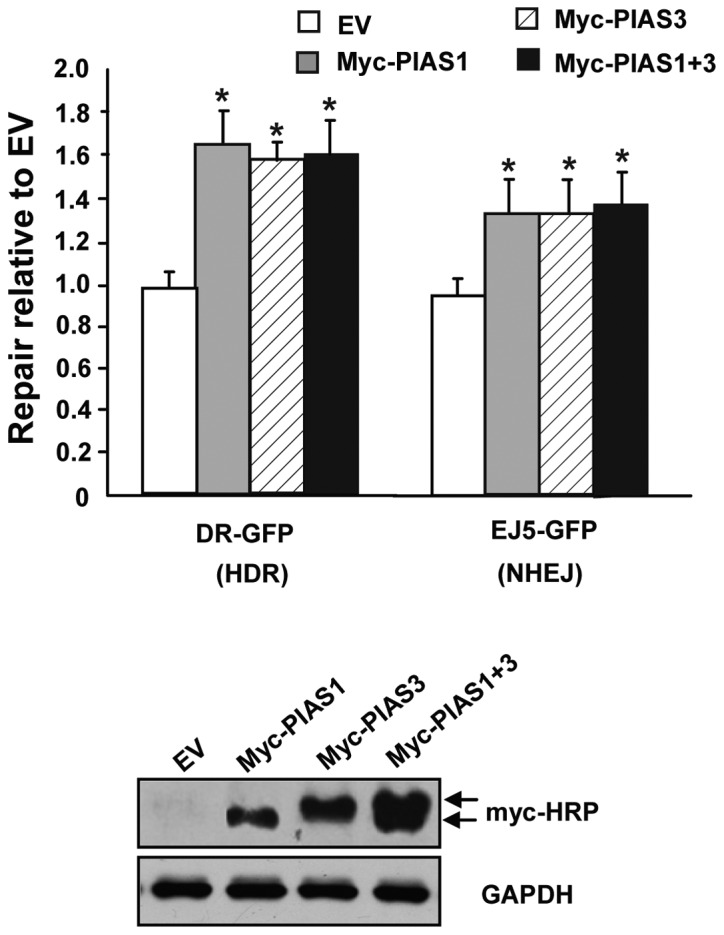
PIAS3 promotes HDR and distal NHEJ. The human 293T cells were transfected with an expression vector for I-SceI, along with a complementation vector for PIAS1, PIAS3, P1AS1 plus PIAS3 or the empty expression vector (EV). Repair is measured as the percentage of GFP^+^ cells. (^*^P<0.001, statistical differences between EV and PIAS1, PIAS3 or P1AS1 plus PIAS3 treatments). Representative western blots of PIAS1 and PIAS3 expression in human 293T cells are shown in the lower panel, with PIAS1 and PIAS3 carrying Myc-HRP. PIAS, protein inhibitor of activated STAT; HDR, homology-directed repair; NHEJ, non-homolous end joining; GFP, green fluorescent protein; HRP, horseradish peroxidase.

Mammalian SUMO E3-ligase PIAS1 was reported to promote the response to DNA DSBs (17). Therefore, other PIAS family members, including PIAS3, may also be involved in DSB repair. A PIAS3 expression vector was transfected into the well-established HR- and NHEJ-mediated DSB repair systems, respectively. PIAS1 was used as a positive control. The overexpression of PIAS1 and PIAS3 resulted in a 1.6-fold increase of GFP^+^ cells in comparison with the empty vector cells (Fig. 3), and the co-transfection of PIAS1 and PIAS3 did not synergistically increase the GFP^+^ cells. This result indicates that PIAS3 promotes HDR and distal-NHEJ, as does PIAS1. PIAS3 and PIAS1 do not have a synergistic effect on HDR and distal NHEJ.

### Overexpression of PIAS3 confers IR resistance

**Figure 4:**
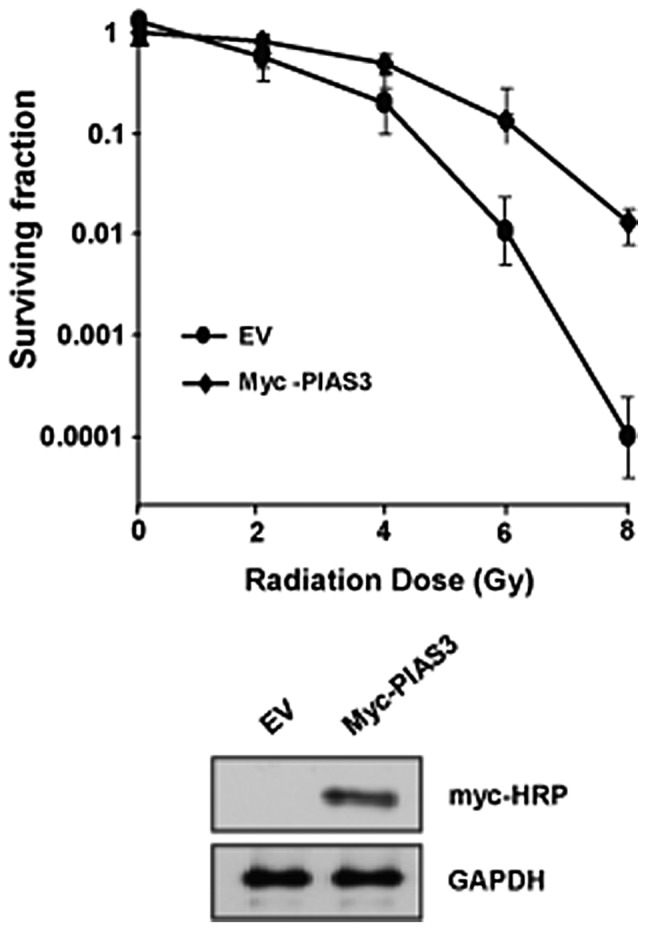
Expression of PIAS3 in HeLa cells increases the cell resistance to IR. The surviving fraction of the empty vector Hela cells and the Hela cells overexpressing PIAS3 following exposure to various doses of irradiation is shown. Each point represents the mean surviving fraction. Error bars indicate the standard deviation of two independent biological samples of one experiment. PIAS, protein inhibitor of activated STAT; IR, ionizing radiation; HRP, horseradish peroxidase; EV, empty expression vector.

The overexpression of PIAS3 resulted in an increase of HR- and NHEJ-mediated DSB repair. PIAS3 was able to upregulate IR resistance. The expression of PIAS3 in the HeLa cells (Fig. 4) increased the cell resistance to IR. PIAS3 plays a significant role in promoting IR resistance, therefore, PIAS3 may be a potentially promising therapeutic approach for cancer treatment.

## Discussion

The PIAS family of proteins was named based on the identification of the founding member, PIAS3, as a repressor of the activity of the STAT3 transcription factor (18). Since then, three additional family members, PIAS1, PIAS2 and PIAS4, have been identified and are characterized by a high degree of sequence conservation throughout the proteins (19). The PIAS proteins have been shown to impact on the function of a number of proteins, but a major process on which all these proteins act is the control of gene transcription. Thus, PIAS proteins may be considered to be transcriptional coregulators. PIAS protein action may be activated or repressed, although the mechanism of action apparently differs depending on the target gene or interacting transcriptional regulator. The other major functional part of PIAS proteins is the SP-RING domain, which is associated with the zinc-binding RING fingers and is most similar to the domains that have been identified in a subclass of ubiquitin E3 ligases (18,20). These somewhat functionally-redundant proteins are structurally associated with ubiquitin and are covalently attached to target proteins by a SUMO-conjugation system consisting of an E1 activating enzyme (SAE1/SAE2), an E2 ligase (Ubc9) and various E3 ligases with differing target-protein specificities (20). The present study identified that PIAS3 not only promotes HR repair, but that it also promotes NHEJ repair. Given the fact that PIAS3 serves as the ligase for protein sumoylation in DSB repair (21), PIAS3 may modulate the sumoylation status of key DDR factors, including CtIP and DNA-PKcs/Ku70/Ku80. However, the molecular mechanisms of PIAS3 in DSB repair require further investigation.

A number of tumor-associated mutations, including ATM, BRCA1, BRCA2, CHK2 and p53, have been identified to be clustered in the HR pathway (19,22–24). Therefore, promoting HR in human tumors may be a highly useful strategy to combat cancer by enhancing the effect of radiation or DSB-inducing chemotherapy. This may be of particular significance to breast cancer therapy, as a significant percentage of hereditary breast cancers carry the BRCA1 or BRCA2 mutations and thus are deficient in HR (22,25,26). Therefore, PIAS3 mimetics are promising candidates for the development of sensitizers for the treatment of BRCA-deficient breast cancers using DNA-damaging chemotherapeutic drugs and radiation. This study serves as a proof-of-principle of targeting SUMO-dependent functions in the development of novel therapeutics, as well as in uncovering the role of SUMO modifications in various cellular functions.

In conclusion, PIAS3 is an enhancer of HR- and NHEJ-mediated DSB repair that increases cell resistance to IR.
